# Mental health, compliance with measures and health prospects during the COVID-19 epidemic: the role of health literacy

**DOI:** 10.1186/s12889-021-11437-w

**Published:** 2021-07-10

**Authors:** Lize Hermans, Stephan Van den Broucke, Lydia Gisle, Stefaan Demarest, Rana Charafeddine

**Affiliations:** 1 Department of Epidemiology and Public Health, Sciensano, Brussels, Belgium; 2 Université Catholique de Louvain, Louvain-la-Neuve, Belgium

**Keywords:** Health literacy, COVID-19, Mental health, Preventive measures

## Abstract

**Background:**

The importance of health literacy in dealing with the COVID-19 epidemic has been emphasized but scarcely addressed empirically. In this study, the association of health literacy with mental health, compliance with COVID-19 preventive measures and health prospects was assessed in a Belgian context.

**Methods:**

Data were extracted from the third of a series of cross-sectional online COVID-related surveys (*n* = 32,794). Data collection took place for 1 week starting the 28th of May 2020. People residing in Belgium and aged 18 years or older could participate. Data were collected on sociodemographic background, health literacy, multimorbidity, mental health (depression, anxiety, sleeping disorder, vitality), knowledge about COVID-19, compliance with COVID-19 measures (hygiene, physical distance, covering mouth and nose on public transport and in places where physical distance cannot be respected), and health prospects (risk for health when returning to normal life and possibility of infection). Prevalence Ratio (PR) of poor mental health, non-compliance with the measures and health prospects in relation to health literacy were calculated using Poisson regressions.

**Results:**

People showing sufficient health literacy were less likely to suffer from anxiety disorders (PR = 0.47, 95% CI = [0.42–0.53]), depression (PR = 0.46, 95% CI = [0.40–0.52]) and sleeping disorders (PR = 0.85, 95% CI = [0.82–0.87]), and more likely to have optimal vitality (PR = 2.41, 95% CI = [2.05–2.84]) than people with low health literacy. They were less at risk of not complying with the COVID-19 measures (PR between 0.60 and 0.83) except one (covering mouth and nose in places where physical distance cannot be respected). Finally, they were less likely to perceive returning to normal life as threatening (PR = 0.70, 95% CI = [0.65–0.77]) and to consider themselves at risk of an infection with COVID-19 (PR = 0.75, 95% CI = [0.67–0.84]). The associations remained significant after controlling for COVID-19 knowledge and multimorbidity.

**Conclusions:**

These results suggest that health literacy is a crucial factor in managing the COVID-19 epidemic and offer a perspective for future studies that target health literacy in the context of virus outbreaks.

**Supplementary Information:**

The online version contains supplementary material available at 10.1186/s12889-021-11437-w.

## Background

On March 12, 2020 the World Health Organization declared the coronavirus disease 2019 (COVID-19) outbreak a pandemic. Since then, governments worldwide have been taking strong action in the fight against the spread of the virus by implementing unprecedented measures. Citizens are asked to frequently wash their hands, keep sufficient distance from others, wear face masks that cover their mouth and nose in designated places, and limit their social contacts. To encourage people to comply with these preventive measures, authorities have used different approaches, ranging from giving simple recommendations on how to protect oneself against the virus, issuing warnings, and calling upon peoples’ sense of responsibility, to imposing legal restrictions and severely penalizing violations. These efforts have only been partially successful, as a significant part of the population does not strictly comply with the recommendations [1]. While to some extent this may be due to forgetfulness, unwillingness to follow rules imposed by authorities, or practical conditions that make strict compliance difficult, non-compliance with preventive measures against COVID-19 may also result from flaws in the communication process and information delivered to the public. Indeed, for instructions to be followed, it is important that people have the ability to easily access them, understand them, accept them, and apply them to their own situation. In other words, they have to be sufficiently *health literate* in this case.

While its role is not always recognized, health literacy may well be a crucial factor in the control of the epidemic [2, 3]. In ordinary times, more than one in three people struggle to access, understand, appraise and use information that is necessary to improve or maintain their health [4, 5]. People who are less educated, who are older, or who have a lower social status are more at risk of showing low health literacy [4]. Yet whereas the positive effect of health literacy on the prevention and self-management of non-communicable diseases has been well established [6], the literature focusing on health literacy in relation to infectious diseases is limited and fragmented [7].

Given the recency of the coronavirus outbreak, the literature on health literacy and COVID-19 is only beginning to emerge [8]. A few studies have been published since the outbreak looking at health literacy skills that are specific to COVID-19 [9] or investigating online searches for information about the pandemic [10]. Based on the literature and theory regarding health literacy, one would expect that people with higher levels of health literacy are more likely to adopt behaviors that may help contain the epidemic, such as handwashing, facial mask wearing and physical distancing. However, the role of health literacy in the compliance with preventive measures against COVID-19 has thus far only marginally been addressed [1, 11]. Being health literate might also reduce the impact of the stress caused by the COVID-19 pandemic on mental health. Healthcare workers who are at the frontline are at high risk of burnout, anxiety, depression and post-traumatic stress disorder [12, 13]. Furthermore, the spread of the coronavirus and the confinement measures that have been taken to prevent this spread may impact the mental health of the general population [12, 14–16]. Indeed, researchers in many countries found that the mental health of the population has deteriorated since the outbreak of the virus [17–22]. So far, the relationship between health literacy and mental health during the COVID-19 pandemic has solely been explored in three studies, the results of which suggest a buffering effect of health literacy [23–25].

The potential adverse effect of low health literacy on the compliance with preventive measures and on mental health may be influenced by the huge media and social media coverage about the pandemic, which some authors refer to as the COVID-19 *infodemic* [26]. As this abundance of often contradictory information is not only confusing, but can also contain misleading or even downright incorrect information, it is even more important for people to carefully consider the information sources and have the necessary skills to distinguish between reliable and unreliable information [27–29].

Finally, health literacy may also affect how people perceive their future health. Research has shown that people with low health literacy are less accurate at risk perception [30, 31]. In the context of COVID-19, it has been reported that low health literacy is associated with lower *fear* of the virus [32], but it is not known whether it also has a positive effect on how people perceive their personal *risk* of contracting the virus or the risk for their health when returning to normal life.

So, while it can be assumed that health literacy is of vital importance to deal with the COVID-19 pandemic in several ways, the evidence to support that view is scarce. This study aimed to address this deficit by assessing the association of health literacy with non-compliance of the preventive measures against COVID-19, mental health, and health prospects. The hypothesis was that people with higher levels of health literacy would better comply with the preventive measures, would exhibit better mental health, and would have more positive health prospects than people with low health literacy.

## Methods

### Participants

To study the impact of the COVID-19 pandemic on the perceived health status and preventive behavior of the population in Belgium, Sciensano, the national institute of health, organized a series of cross-sectional online surveys. The first COVID-19 health survey was launched 3 weeks after the start of a nation-wide confinement imposed by the government following the outbreak of the pandemic (2nd of April), the second survey took place 2 weeks later (16th of April), and the third one on the 28th of May 2020, when the confinement was partially eased. Every survey remained online for 1 week. The launch of the surveys was announced in the press as well as on the website and social media of Sciensano and of other organizations (health insurance organizations, community centers…). Participation was based on snowball sampling [33], in the sense that respondents were asked to share the link to the survey with their family, friends and acquaintances residing in Belgium and aged 18 years and older. People younger than 18 or not living in Belgium could not participate and thus were excluded from the study. Participants who had agreed in a given survey to take part in the next one received an invitation through the e-mail address they provided. All methods were carried out in accordance with relevant guidelines and regulations. The survey was approved by the ethical committee of the Ghent University Hospital (BC-07544) and informed consent was obtained from all participants.

Since health literacy was only assessed in the third survey, data were extracted from this survey. A total of 37,409 people visited the website of the survey but only data from people who provided information on their age, gender, zip code and educational level were retained. The final sample consisted of 32,794 participants.

### Measures

An overview of the measures that were used for the study, including all the questions and answer categories, can be found in the supplementary materials.

#### Health literacy

Health literacy was assessed using the revised European Health Literacy Survey Questionnaire (HLS_19_-Q12), developed by the international M-POHL Consortium as a revised version of the European Health Literacy Survey Questionnaire. The latter was developed for the European Health Literacy study (HLS-EU) and has been translated and used in a large number of countries [34]. The derived HLS_19_-Q12 has been validated in Europe [35] and consists of 12 items that reflect the dimensions of health literacy proposed in the model of Sørensen et al. [36], assessing how easy or difficult it is for people to find, understand, appraise health information and act accordingly. People with low health literacy were identified using the method described by Sørensen et al. [4] and Pelikan et al. [34]. In accordance with the European Health Literacy Survey, the score for health literacy was standardized into a scale from 0 to 50 (HL = (mean − 1)*(50/3)), and on this basis two categories of health literacy were defined: low health literacy (score of 0–33) and sufficient health literacy (score of > 33–50).

#### Outcome measures

Three outcomes were examined in this study: mental health, non-compliance with preventive measures and health prospects.

##### Mental health

The presence of anxiety and depression was assessed using the Generalized Anxiety Disorder Scale (GAD-7) and the Patient Health Questionnaire (PHQ-9), respectively. The presence of a sleeping disorder was assessed by the Symptom Checklist – 90 – Revised (SCL-90-R) [37]. Optimal vitality was assessed using the four items of the RAND SF-36-V2 [38].

##### Non-compliance with the preventive measures

The participants’ compliance with the preventive measures to reduce the spread of the virus was assessed using a simple question with “low respect”, “partial respect” and “strict respect” as answer categories for each of the following recommended behaviors: hand hygiene (washing hands regularly, coughing in elbow, ...), maintaining a distance of at least 1.5 m from people (except for the people you live with), covering mouth and nose with a face mask, scarf or bandana on public transport, and covering mouth and nose in places where a distance of 1.5 m cannot be guaranteed.

##### Health prospects

Health prospects were assessed by two questions to be scored on Likert-type scales: (1) the perceived health risk of returning to normal life, scored on a 4-point Likert-type scale ranging from very low to very high risk, and (2) the likelihood of a future COVID-19 infection, scored on a 5-point Likert-type scale ranging from very unlikely to very likely.

#### Covariates

Multimorbidity was measured with the question “In the 12 months prior to 13 March, did you have any of the following diseases or conditions?” (Yes/No) (followed by a list of 16 frequent chronic diseases). Knowledge about COVID-19 was measured by six direct questions asking whether the respondent considered him/herself sufficiently informed about the following issues: preventive measures against a coronavirus (COVID-19) infection, symptoms of a coronavirus (COVID-19) infection, the treatment of a coronavirus (COVID-19) infection, the spread of the coronavirus, the easing of the containment and quarantine measures, and the availability of shops and services during corona. All questions were scored on a 2-point scale (sufficiently informed - not sufficiently informed) and summed.

### Statistical analyses

Following an inspection of descriptive statistics for all the variables, Poisson regressions modeling were used to study the association of each of the three outcomes (mental health, the non-compliance with preventive measures and the health prospects) with health literacy during the COVID-19 pandemic. Two models were estimated for each outcome variable. The first model controlled for sex (male/female), age groups (18–24, 25–34, 35–44, 45–54, 55–64, 65+), and education (secondary education or lower, higher than secondary education). Educational level is an important confounder due to its association with health literacy and with the studied outcomes. For the second model, multimorbidity (0 disease, 1–2 diseases, 2 or more diseases) and knowledge about COVID-19 (low knowledge versus sufficient knowledge) were added. Multimorbidity was included in the model because people’s reaction to the COVID-19 epidemic may vary if a person had a chronic disease. Knowledge about COVID-19 was included to control for knowledge in relation to literacy. Missing values for the explanatory variables were treated as a separate category.

Post-stratification weights were used in all the analyses to align the sample to the Belgian population in terms of age, sex, province and educational level. Confidence intervals were calculated at the 95% level. The analyses were performed in STATA 16 using appropriate svy commands.

## Results

Table 1 presents the distribution of the participants’ characteristics in terms of sociodemographic and explanatory variables. The table includes the weighted percentage of the missing values for each explanatory variable. The results showed that 23% of the general population had low levels of health literacy while 56% had sufficient levels. If the missing values for health literacy were excluded (21%), the percentages would be 29% and 71%, respectively.

**Table 1:** Distribution of the study population (*N* = 32,794) by sociodemographic covariates and explanatory variables

	All	Health literacy (weighted %)		
	(weighted %)	Low	Sufficient	Missing
**Gender**				
Male	49.05	23.91	53.68	22.42
Female	50.95	21.37	58.73	19.90
**Age group (years)**				
18–24	13.75	28.36	38.67	32.97
25–34	15.70	26.81	48.13	25.06
35–44	15.73	22.19	55.73	22.08
45–54	16.64	22.15	59.30	18.54
55–64	16.02	20.84	62.86	16.29
65+	22.16	18.01	66.19	15.79
**Education**				
Secondary education or lower	67.15	23.91	52.93	23.16
Higher education	32.85	19.97	63.04	16.99
**Multimorbidity**				
0 disease	51.96	21.97	57.55	20.48
1–2 diseases	25.18	23.69	61.02	15.29
2 or more diseases	12.71	26.18	59.38	14.44
Missing	10.15	18.80	33.83	47.38
**Knowledge about COVID-19**				
Limited	29.63	42.14	43.08	14.78
Sufficient	57.54	16.32	73.58	10.09
Missing	12.82	5.73	8.90	85.37
**All**	100	22.62	56.25	21.14

Table 2 presents the prevalence of the outcome measures in the general population and by health literacy status, and includes the number of participants for each outcome measure. At this stage of the epidemic, almost 16% of the participants were found to suffer an anxiety disorder, and 15% a depression, while 72% experienced a sleeping disorder and only 16% met the optimal vitality threshold. These percentages were higher for respondents with a low level of health literacy compared to those with sufficient health literacy.

**Table 2:** Prevalence of mental health, compliance with measures and health prospects in the general population

	All	Health literacy (weighted %)		
	(weighted %)	Low	Sufficient	Missing
**Mental health**				
Anxiety disorder (*n* = 31,226)				
Yes	16.06	27.07	11.32	17.23
No	83.94	72.93	88.68	82.77
Depression (*n* = 30,585)				
Yes	15.16	26.43	10.53	15.58
No	84.84	73.57	89.47	84.42
Sleeping disorder (*n* = 31,519)				
Yes	72.01	81.59	68.57	70.63
No	27.99	18.41	31.43	29.37
Optimal vitality (*n* = 31,759)				
Yes	15.75	7.22	19.70	14.06
No	84.25	92.78	80.30	85.94
**Compliance with measures**				
Hygiene measures (*n* = 30,027)				
Yes	81.65	76.76	84.65	76.13
No	18.35	23.24	13.35	23.87
Keeping physical distance (*n* = 29,983)				
Yes	74.47	68.84	77.89	68.27
No	25.53	31.16	22.11	31.73
Covering mouth and nose on public transport (*n* = 20,351)				
Yes	94.98	93.50	96.15	91.47
No	5.02	6.50	3.85	8.53
Covering mouth and nose in places where physical distance cannot be respected (*n* = 28,501)				
Yes	72.77	70.06	75.07	65.87
No	27.23	29.94	24.93	34.13
**Health prospects**				
High risk for health when returning to normal life (*n* = 29,504)				
Yes	24.68	30.06	22.76	23.45
No	75.32	69.94	77.24	76.55
Likely to be infected with COVID-19 (*n* = 29,358)				
Yes	18.48	23.90	16.73	16.30
No	81.52	76.10	83.27	83.70

The prevalence of non-compliance of preventive measures varied depending on the measure considered. Only 5% of the respondents indicated that they did not strictly comply with measure of covering their mouth and nose on public transport, whereas 27% indicated they did not strictly comply with the measure of covering mouth and nose in places where physical distance could not be guaranteed. Maintaining a physical distance of at least 1.5 m was not strictly followed by 26% of the respondents, and hygiene measures (handwashing) by 18%. Again, the percentages of non-complying with preventive measures were higher for respondents with low health literacy.

With respect to health prospects, approximately one out of four respondents thought that returning to normal life entailed a high risk for their own health, and one out of five thought they were likely to become infected with the coronavirus in the coming months. These percentages were again higher for respondents with low health literacy.

Table 3 presents the prevalence ratio (PR) of the outcomes in relation to health literacy. Health literacy was significantly associated with mental health during the COVID-19 pandemic. Specifically, people with sufficient health literacy were less likely to suffer from anxiety disorders, depression and sleeping disorders than those with low health literacy, and were more than twice as likely to have an optimal level of vitality. The association remained significant after controlling for knowledge about COVID-19 and multimorbidity. Health literacy was also significantly associated with non-compliance of the COVID-19 measures, with the risk of not adhering being lower for participants with sufficient health literacy than for those with low health literacy for all but one measure (covering mouth and nose in places where the physical distance cannot be respected). The associations remained significant after controlling for knowledge about COVID-19 and multimorbidity. We also found significant associations between health literacy and health prospects, in the sense that respondents with sufficient health literacy were less likely to perceive returning to normal life as risky than those with low health literacy. They were also less likely to consider themselves at risk of an infection with COVID-19 in the future. Again, the results remained significant after controlling for knowledge about COVID-19 and multimorbidity.

**Table 3:** Mental health, non-compliance with the measures and health prospects during the COVID-19 pandemic in people with low (baseline) versus sufficient health literacy

		Health literacy PR (95% CI) Model 1	Health literacy PR (95% CI) Model 2
Mental health	Anxiety disorder	0.47 (0.42–0.53)***	0.56 (0.49–0.63)***
	Depression	0.46 (0.40–0.52)***	0.54 (0.47–0.62)***
	Sleeping disorder	0.85 (0.82–0.87)***	0.88 (0.85–0.91)***
	Optimal vitality	2.41 (2.05–2.84)***	2.13 (1.81–2.50)***
Non-compliance with…	Hygiene measures	0.74 (0.66–0.83)***	0.76 (0.68–0.86)***
	Physical distance	0.83 (0.76–0.91)***	0.86 (0.78–0.94)**
	Covering mouth and nose on public transport	0.60 (0.47–0.77)***	0.64 (0.50–0.83)**
	Covering mouth and nose in places where physical distance cannot be respected	0.92 (0.84–1.00)	0.92 (0.84–1.01)
Health prospects	High risk for health when returning to normal life	0.70 (0.65–0.77)***	0.79 (0.73–0.87)***
	Likely to be infected with COVID-19	0.75 (0.67–0.84)***	0.81 (0.72–0.91)***

Poisson regressions. Model 1 includes age group, gender, education and health literacy. Model 2 includes age group, gender, education, health literacy, knowledge COVID-19 and multimorbidity. *PR* Prevalence ratio, *CI* Confidence interval

**p < .01, ***p < .001

## Discussion

Health literacy is considered a critical concept for public health, the importance of which is attested by the large body of scientific literature that relates health literacy to medication and health service use, adherence to disease self-management, use of preventive services, participation in screening and vaccination programs, and engagement in health promoting behaviors [39]. Yet despite this agreed-upon importance, the role of health literacy in adopting preventive measures against COVID-19 has scarcely been examined empirically. This survey study, involving a large sample of the Belgian population, investigated whether health literacy is related to the likelihood of people to adopt behaviors that help to contain the epidemic, notably hand hygiene, physical distancing, and wearing a face mask or other protection covering nose and mouth on public transport and in places where a physical distance of at least 1.5 m cannot be guaranteed. The results indicate that people with low levels of health literacy are less likely to comply with the preventive behavioral measures. Moreover, they also report more mental health problems, and have a more negative perspective of future health than people with sufficient health literacy.

The results of this study showed that 29% of the Belgian population have low levels of health literacy. This is comparable to previous Belgian population surveys: in the Health Interview Survey 2018 [40] 33% of the respondents were found to have low levels of health literacy. People with sufficient health literacy were less likely not to comply with the preventive measures. Furthermore, those with sufficient health literacy were less likely to suffer from a depression, anxiety or sleeping disorder. This is in agreement with Nguyen et al. [25] who reported lower odds of depression with higher levels of health literacy. Furthermore, Duplaga et al. [23] showed that higher health literacy was associated with lower levels of future anxiety during the COVID-19 pandemic. As such, these findings confirm the importance of health literacy in people’s reaction to the pandemic, and suggest that health literacy should be taken in account in order to address (the consequences of) the pandemic effectively.

Taking health literacy in account implies that the communication about COVID-19 and about the preventive measures needs to be adapted to the literacy needs of the people. According to Okan, Sørensen and Messer [41] this may involve: presenting information in a comprehensible manner and adapting the type and amount of communication to your audience (e.g. using animations for people with low health literacy); clarifying that evidence on the virus is advancing and new information might emerge that may lead to recommendations being modified; communicating new information on the virus and if needed correcting earlier messages. It also involves countering false or misleading information about COVID by encouraging people to cross-check its accuracy and credibility, to check the information sources and verify the information by consulting a second source, to consult trusted health professionals about information that is suspect, and to not share information that has not been fact-checked [41]. Given the unprecedented coverage of the pandemic in traditional mass media and on social media, which often adds to the confusion [26], it is an important task for both conventional and social media to ensure that the information that is provided on COVID-19 and on the precautionary measures is correct [42], comprehensive, transparent, consistent, supportive and easy to understand [42–44].

In addition to calling for an adaptation and improvement of the communication about the pandemic and the response to it, our findings also emphasize the continuing need to improve the health literacy of the population [45], so as to enable people to make sound health decisions in the context of the pandemic and beyond. Such an improvement is not only a task of the health sector, but requires action at different levels, including governmental agencies and the media [46]. Such actions could include, amongst others, the development of guidelines to ensure better health communication comprehensible to people with low health literacy, the inclusion of health literacy in the training curricula of health workers, implementing health literacy programs in school settings [47], the creation and strengthening of health literacy-friendly settings, and the development of policies for health literacy at the local, national and international level.

This study is not without its limitations. As the survey was only made available online and a snowball sampling technique was used to recruit participants, the results may not be representative of the entire population, despite the fact that a weighing of the data for age, sex and education was applied to improve representativeness. Also, a relatively large number of respondents (approximately 20%) did not complete or only partially completed the questions on health literacy. On the other hand, the percentage of respondents with a sufficient level of health literacy is comparable to that of previous population surveys in Belgium [48]. Lastly, as the study design was cross-sectional, the causality of the relationship between health literacy and the outcome variables cannot be inferred.

Despite these limitations, however, the results of this study are sufficiently robust to assert the importance of health literacy as a determinant of people’s reactions to COVID-19. While offering evidence that health literacy should be taken in account to more efficiently curb the pandemic, they also offer a perspective for future studies that target health literacy in the context of virus outbreaks.

## Supplementary Information

**Additional file 1: Supplementary 1.** Overview of the creation of the indicators based on the questions and answer categories in the third COVID-19 health survey.

## Data Availability

The dataset used and/or analysed during the current study is available from the corresponding author following a request to the Health Interview Survey team at Sciensano and a signature of a Data Transfer Agreement.

## Abbreviations

CI: Confidence interval
COVID-19: Coronavirus disease 2019
GAD-7: Generalized Anxiety Disorder Scale
HLS_19_-Q12: European Health Literacy Survey Questionnaire
PHQ-9: Patient Health Questionnaire
PR: Prevalence Ratio
SF-36: Short Form 36
