# Supplementary material for: Mental health, compliance with measures and health prospects during the COVID-19 epidemic: the role of health literacy
Source: BMC Public Health. 2021 Jul 10;21:1365. doi: 10.1186/s12889-021-11437-w (PMC8270766; doi:10.1186/s12889-021-11437-w)
Supplement: Supplementary file 1 — Additional file 1: Supplementary 1. Overview of the creation of the indicators based on the questions and answer categories in the third COVID-19 health survey. [file 12889_2021_11437_MOESM1_ESM.docx]

***mental health, compliance with measures and health prospects during the covid-19 epidemic: the role of health literacy***

Hermans Lize^1^, Van den Broucke Stephan^2^, Gisle Lydia^1^, Demarest Stefaan^1^, Charafeddine Rana^1^

^1^ Sciensano, Scientific Direction Public Health and Epidemiology, Belgium

^2^ Université Catholique de Louvain, Louvain-la-Neuve, Belgium

**Corresponding author:**

Lize Hermans

Sciensano

Scientific Direction Public Health and Epidemiology

Juliette Wytsmanstraat 14

B-1050 Belgium

[lize.hermans@sciensano.be](mailto:lize.hermans@sciensano.be)

Supplementary 1. Overview of the creation of the indicators based on the questions and answer categories in the third COVID-19 health survey.

| **Indicator** | **Questions** | **Answer categories** | **Indicator categories** |
| --- | --- | --- | --- |
| Health literacy | It is not always easy to obtain understandable, reliable and useful information on health-related topics. With the following questions we would like to know which tasks related to the processing of health information are more or less easy or difficult. On a scale from very easy to very difficult, how easy would you say it is ...   - to find out where to get professional help (for instance, from a doctor, nurse, pharmacist, psychologist, ...) when you are ill? - to understand information about what to do in a medical emergency? - to judge the advantages and disadvantages of different treatment options? - to act on advice from your doctor or pharmacist? - to find information on how to handle mental health problems (stress, depression or anxiety)? - to understand information about recommended health screenings or examinations (for instance, colorectal cancer screening, blood sugar test)? - to judge if information on unhealthy habits, such as smoking, low physical activity or drinking too much alcohol, are reliable? - to decide how you can protect yourself from illness using information from the mass media (for instance, newspapers, TV or Internet)? - to find information on healthy lifestyles such as physical exercise or healthy nutrition? - to understand advice concerning your health from family or friends? - to judge how your housing conditions may affect your health and well-being? - to make decisions to improve your health and well-being? | 1. Very easy  (score = 4) 2. Fairly easy  (score = 3) 3. Fairly difficult  (score = 2) 4. Very difficult  (score = 1) | Scores were summed and divided by 12 to obtain the mean score. The following formula was applied to normalize the mean score on a scale of 0 to 50: *index = (mean-1)*(50/3)*.   1. Sufficient health literacy (>33) 2. Low health literacy  (0-33) |
|  |  |  |  |
| Anxiety | Were you bothered by the following problems over the last 2 weeks?   - Feeling nervous, anxious or on edge - Not being able to stop or control worrying - Worrying too much about different things - Trouble relaxing - Being so restless that it is hard to sit still - Becoming easily annoyed or irritable - Feeling afraid as if something awful might happen | 1. No, not at all (score = 0) 2. Yes, several days (score = 1) 3. Yes, more than half the time (score = 2) 4. Yes, nearly every day (score = 4) | The scores were summed (range 0-27) and case definition established at 10+.   1. Yes (≥ 10) 2. No (0-9) |
|  |  |  |  |
| Depression | Were you bothered by the following problems over the last 2 weeks?   - Little interest or pleasure in doing things - Feeling down, depressed, or hopeless - Trouble falling or staying asleep, or sleeping too much - Feeling tired or having little energy - Poor appetite or overeating - Feeling bad about yourself - or that you are a failure or have let yourself or your family down - Trouble concentrating on things, such as reading the newspaper or watching television - Moving or speaking so slowly that other people could have noticed? Or the opposite: being so fidgety or restless that you have been moving around a lot more than usual - Thoughts that you would be better off if you were no longer alive | 1. No, not at all (score = 0 ) 2. Yes, several days (score = 1) 3. Yes, more than half the time (score = 2) 4. Yes, nearly every day (score = 3) | Based on the diagnostic algorithms of PHQ-9 for major depression and other depression^[[1]](#footnote-1)^   1. Yes (major depression or other depression present) 2. No (neither major depression or other depression present) |
|  |  |  |  |
| Sleeping disorder | How much during the last 2 weeks...   - did you have trouble falling asleep? - did you waken too early in the morning? - was your sleep restless or disturbed? | 1. Never (score = 0) 2. Rarely (score = 1) 3. Sometimes (score = 2) 4. Often (score = 3) 5. All the time (score = 4) | The scores were summed (range 0-12), then divided by 3 (nr of items) and rounded (range 0-4) Case definition was established at 2+.   1. Yes (≥2) 2. No (0-1) |
|  |  |  |  |
| Optimal vitality | How much during the last 2 weeks...   - did you feel worn out? - did you feel tired? - did you feel full of life? - did you have a lot of energy? | 1. Never (score = 1 ) 2. Rarely (score = 2) 3. Sometimes (score = 3) 4. Often (score = 4) 5. All the time (score = 5) | 100*(mean(6-Q1, 6-Q2, Q3, Q4)-1)/4); cut-off = +1 standard deviation   1. Yes (>78) 2. No (0-78) |
|  |  |  |  |
| Non-compliance with hygiene measures | To what extent do you respect the following measures since their introduction?   - Hygiene measures (washing hands regularly, coughing in elbow, ...) | 1. Strict respect 2. Partial respect 3. Low respect | 1. Non-respect  (answer categories 2&3) 2. Strict respect (answer category 1) |
|  |  |  |  |
| Non-compliance with physical distance | To what extent do you respect the following measures since their introduction?   - Maintain a distance of at least 1.5 m from people (outside the people you live with) | 1. Strict respect 2. Partial respect 3. Low respect | 1. Non-respect  (answer categories 2&3) 2. Strict respect (answer category 1) |
|  |  |  |  |
| Non-compliance with covering mouth and nose on public transport | To what extent do you respect the following measures since their introduction?   - Cover mouth and nose with a face mask, scarf or bandana on public transport | 1. Strict respect 2. Partial respect 3. Low respect | 1. Non-respect  (answer categories 2&3) 2. Strict respect (answer category 1) |
|  |  |  |  |
| Non-compliance with covering mouth and nose in places where physical distance cannot be respected | To what extent do you respect the following measures since their introduction?   - Cover mouth and nose with a face mask, scarf or bandana on other places where a distance of at least 1.5 m from other people cannot be guaranteed | 1. Strict respect 2. Partial respect 3. Low respect | 1. Non-respect  (answer categories 2&3) 2. Strict respect (answer category 1) |
|  |  |  |  |
| High risk for health when returning to normal life | Returning to my normal life entails... | 1. A low risk 2. A rather limited risk 3. A rather high risk 4. A very high risk | 1. High risk  (answer categories 3&4) 2. Low risk (answer categories 1&2) |
|  |  |  |  |
| Likely to be infected with COVID-19 | According to you, how likely is it that you'll be infected with the coronavirus (COVID-19) in the next few months? | 1. Very unlikely 2. Unlikely 3. Neutral 4. Quite likely 5. Very likely | 1. Likely  (answer categories 4&5) 2. Unlikely (answer categories 1,2&3) |
|  |  |  |  |
| Multimorbidity | \| In the 12 months prior to 13 March, did you have any of the following diseases or conditions?  A list of 16 frequent chronic diseases:  1. Asthma (allergic asthma included)  2. Chronic bronchitis, chronic obstructive pulmonary disease, emphysema  3. Myocardial infarction  4. Coronary heart disease (angina pectoris)  5. Other serious heart disease  6. High blood pressure (hypertension)  7. Stroke (cerebral hemorrhage, cerebral thrombosis) or its consequences  8. Other chronic neurological disease  9. Rheumatoid arthritis (inflammation of the joints)  10. Osteoarthritis (arthrosis, joint degeneration)  11. Diabetes  12. Cancer (malignant tumor, also including leukemia and lymphoma)  13. Serious gloom or depression for a period of at least 2 weeks  14. Severe anxiety disorder  15. Chronic kidney disease  16. Suppression of immune defense (also due to the use of chemotherapy) \| \| --- \| | 1. Yes 2. No | 1. 0 disease 2. 1-2 diseases 3. 2 or more diseases |
|  |  |  |  |
| Knowledge about COVID-19 | Can you indicate whether you are sufficiently informed on ...   - Preventive measures against a coronavirus (COVID-19) infection - Symptoms of a coronavirus (COVID-19) infection - The treatment of a coronavirus (COVID-19) infection - How the coronavirus (COVID-19) spreads - The loosening of the containment and quarantine measures - Shops and services availability | 1. Sufficiently informed (score = 1) 2. Not sufficiently informed (score = 0) 3. No opinion  (score = 0) | The scores were summed.   1. Limited knowledge (0-5) 2. Sufficient knowledge (≥5) |
|  |  |  |  |
| All variables are categorical. |  |  |  |

1. Major Depressive Syndrome if Q#1 or 2 >0 and five or more of Q#1-9 are at least “More than half the days” (count Q#9 if present at all) .

   Other Depressive Syndrome if Q#1 or 2 >0 and two, three, or four of Q#1-9 are at least “More than half the days” (count Q#9 if present at all). [↑](#footnote-ref-1)
